# Anti-Tetanus Vaccination Is Associated with Reduced Occurrence and Slower Progression of Parkinson’s Disease—A Retrospective Study

**DOI:** 10.3390/biomedicines12122687

**Published:** 2024-11-25

**Authors:** Ariel Israel, Eli Magen, Eytan Ruppin, Eugene Merzon, Shlomo Vinker, Nir Giladi

**Affiliations:** 1 Leumit Research Institute, Leumit Health Services, Tel-Aviv 6473817, Israel; emarzon@leumit.co.il (E.M.);; 2 Department of Epidemiology and Preventive Medicine, School of Public Health, Faculty of Medical & Health Sciences, Tel Aviv University, Tel-Aviv 6997801, Israel; 3 Medicine A Department, Assuta Ashdod University Medical Center, Ben Gurion University of the Negev, Beer Sheva 8410501, Israel; 4 Cancer Data Science Laboratory, National Cancer Institute, National Institutes of Health, Bethesda, MD 20814, USA; eytan.ruppin@nih.gov; 5 Adelson School of Medicine, Ariel University, Ariel 4070000, Israel; 6 Department of Family Medicine, Faculty of Medical & Health Sciences, Tel-Aviv 6997801, Israel; 7 Brain Institute, Tel-Aviv Sourasky Medical Center, Faculty of Medical & Health Sciences, Sagol School of Neurosciences, Tel Aviv University, Tel-Aviv 6997801, Israel; nirg@tlvmc.gov.il

**Keywords:** Parkinson’s disease, retrospective study, tetanus–diphtheria vaccination, *Clostridium tetani*

## Abstract

**Background:** Parkinson’s disease (PD) is a neurodegenerative disorder that progressively damages the autonomic and central nervous systems, leading to hallmark symptoms such as resting tremor, bradykinesia, and rigidity. Despite extensive research, the underlying cause of PD remains unclear, and current treatments are unable to halt the progression of the disease. In this retrospective study, based on historical electronic health records (EHR) from a national health provider covering the period from 2003 to 2023, we investigated the impact of vaccination and medication purchases on PD occurrence and severity. **Methods:** Using a case–control design, we compared the vaccination histories of 1446 PD patients with 7230 matched controls to assess the association between vaccination and PD onset. Additionally, we explored statistical associations between vaccination, medication purchases, and PD severity over an average of 9 years of follow-up, utilizing a machine learning algorithm to quantify disease severity based on annual antiparkinsonian medication purchases. **Results:** Our analysis revealed a significant reduction in PD occurrence following tetanus–diphtheria (Td) vaccination, with an adjusted odds ratio of 0.17 (95% CI [0.04, 0.70]) for PD onset within 5 years post-vaccination. Furthermore, a time-dependent relationship was identified between the duration since vaccination and both the rate of PD onset and disease progression. Notably, we observed that antimicrobial treatments significantly influenced disease severity, consistent with the antibiotic sensitivity profile of *Clostridium tetani*. **Conclusions:** These findings support the hypothesis that tetanus vaccination and/or *C. tetani* eradication may reduce PD occurrence and slow its progression, suggesting promising directions for future research in PD prevention and treatment.

## 1. Introduction

Parkinson’s disease (PD) is a neurodegenerative disorder affecting over six million individuals worldwide [1]. PD is characterized by the progressive loss of neurons throughout the peripheral autonomic and central nervous systems, with characteristic loss of dopaminergic neurons within the Substantia Nigra. The current diagnosis of PD is based on the presence of resting tremor, bradykinesia, rigidity, and postural response abnormalities, which are tightly associated with dopaminergic loss. PD is a multifactorial disease, with both genetic and environmental factors contributing to its development and progression. The etiology of PD remains poorly understood, although aging, genetic, and environmental factors have been identified as risk factors [2]. Currently, only exercise has shown an effect on disease progression, and no approved treatment targets the basic mechanism of the disease or its direct cause.

Clostridia are Gram-positive obligate anaerobe bacteria that are prevalent in the environment. Several members of the Clostridium species produce highly potent toxins [3]. Notably, *C. tetani* can produce tetanus neurotoxin, the causative agent of tetanus [4,5]. In tetanus, the neurotoxin is internalized into signaling endosomes and transported retrogradely to the neuronal soma, interfering with the release of neurotransmitters, notably glycine, and gamma-aminobutyric acid, blocking inhibitory impulses [6]. Lacking inhibitory neurotransmission, stimulation of motor neurons increases, producing rigidity, unopposed muscle contraction, and spasm [7].

To protect from the severe risks associated with tetanus disease, the combined Tetanus and diphtheria toxoid (TD) vaccine is routinely administered to infants, children, and adolescents as part of the standard immunization programs [8,9]. Adults usually receive anti-tetanus booster vaccination upon clinical indication when they present in the clinic with a wound susceptible to being infected with *C. tetani*, and there is no record of vaccination in the last ten years (or five years in a dirty wound at high risk for contamination). In a serologic survey performed in the United States between 1988 and 1994, fully protective levels of anti-tetanus and anti-diphtheria antibodies were detected in 91% of individuals aged 6 to 11 years but in only 47% of individuals 20 years or older [10,11]. Interestingly, PD happens to be diagnosed at the late adult age and is uncommon before 50 [1], when tetanus antibody protection acquired from childhood and adolescence vaccination loses its effectiveness [10].

Recent studies have revealed significant differences in the gut microbiota composition between patients with Parkinson’s disease (PD) and healthy controls [12]. Notably, alterations were observed for Clostridia species [13]. *C. tetani* has been isolated in the feces of adult individuals [14], suggesting it might be a common host of the human gut. Given its potential to cause synaptic dysfunction, clostridium neurotoxin might play a role in the neurodegenerative process responsible for PD.

In this study, we investigated the hypothesis that *C. tetani* may be involved in the pathogenesis of PD. To explore this, we conducted a large-scale cohort study to determine whether the vaccination history of patients diagnosed with Parkinson’s disease (PD) differs from that of healthy controls and whether medications that affect *C. tetani* proliferation influence disease severity. If a *C. tetani* neurotoxin contributes to the pathogenesis of PD, we would expect an inverse correlation between tetanus toxoid vaccination and both the incidence and the severity of PD. Additionally, antimicrobial treatment that kills *Clostridium* or impairs its proliferation would be expected to reduce PD severity.

## 2. Materials and Methods

### 2.1. Ethics Statement

The Institutional Review Board (IRB) of Leumit Health Services gave ethical approval for this work (LEU-23-0036), with a waiver of informed consent since data were analyzed retrospectively and anonymously.

### 2.2. Study Design

This research was conducted as an observational cohort study in Leumit Health Services (LHS), one of the four national health providers in Israel, providing comprehensive healthcare services to approximately 720,000 members. All Israeli citizens are entitled to comprehensive health insurance and receive a standardized package of health services and medications, as defined by the national “Health Basket” committee. LHS operates a centralized electronic health records (EHR) system with over two decades of meticulously maintained information on patient demographics, medical diagnoses, healthcare encounters, laboratory test results, and records of prescribed and purchased medications. Diagnoses are documented during medical encounters by the treating physicians using the International Classification of Diseases, Ninth Revision (ICD-9). Diagnoses can be marked as chronic when they pertain to a chronic condition, and these can be updated or closed by the treating physicians during subsequent patient encounters. The reliability of these chronic diagnosis records in our registry has been previously validated, demonstrating high accuracy [15].

Eligibility for inclusion in the study was defined as any past or current LHS member with at least five years of LHS membership between years 2003 and 2023. Data extraction was carried out from the LHS central data warehouse in February 2024, encompassing diagnoses, results of laboratory tests, and medication purchases recorded up to 31 December 2023.

### 2.3. Cohort Definition

The study cohort comprises eligible PD patients diagnosed for the first time between ages 45 and 75 alongside a control group matched at a 5:1 ratio of individuals with no documented PD. PD patients were identified by the presence of ICD-9 coded 332 diagnoses “Parkinson’s Disease” in the EHR if recorded by a neurologist or a movement disorder specialist or recorded by any physician if accompanied by purchase of antiparkinsonian medications (listed in Table S7) over a period of more than six months. In order to avoid confusion with overlapping conditions, individuals with a diagnosis indicative of secondary parkinsonism, schizophrenia, pituitary adenoma, restless legs syndrome, or cerebrovascular accident were excluded from the study (diagnoses listed in Table S8), as well as individuals with prior purchase of an antipsychotic medication susceptible to induce parkinsonism (medications listed in Table S9).

Controls were precisely matched to the PD patients based on gender, socio-economic status category, and the year of initial enrollment in Leumit Health Services (LHS). For each PD patient, five control individuals were chosen who met these matching criteria and whose birth dates were closest to that of the PD patient, ensuring no individual was duplicated within the cohort.

### 2.4. Diphtheria-Tetanus Toxoid Vaccinations

We looked for anti-tetanus vaccinations in the medical history of patients from the study cohort, using both vaccine purchases recorded by LHS pharmacies and vaccine administration records documented in the EHRs. Most anti-tetanus vaccinations performed in LHS for patients in the cohort were with dT IMOVAX from Sanofi-Pasteur, adult dose, 0.5 mL.

### 2.5. Data Preparation

Data were extracted from electronic health records and prepared for analyses using scripts developed by Leumit Research Institute in Python 3.11 with Pandas library and T-SQL queries. Prior to analysis, patients’ data were deidentified, and the patient ID number was replaced with an identifier internal to the study.

### 2.6. Statistical Analyses

**Figure 1:**
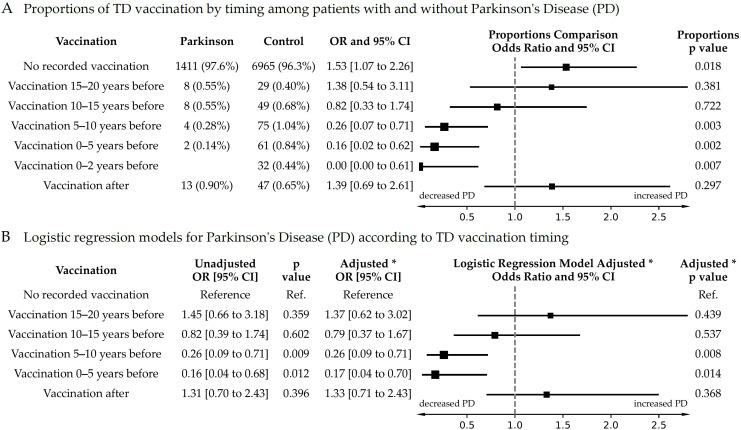
Parkinson’s disease risk according to tetanus–diphtheria (TD) vaccinations with regards to the index date. * Adjusted model is adjusted for age, gender, and smoking status.

Statistical analyses were performed in R version 4.3. Unless specified otherwise, Fisher’s exact test was used to compare categorical variables, including the comparison displayed in Figure 1A, and the two-tailed two-sample *t*-test to compare continuous variables across groups. Conditional logistic models were fit to assess the association of vaccination timing and covariates across matched groups for Figure 1B. Linear regression models were fit to assess the relationship between linear variables such as disease severity and relative disease severity and explanatory variables. All linear regression analyses are adjusted for age, gender, and smoking status. We specifically evaluated the impact of medication purchases from several drug classes, treated as a binary variable indicating whether a purchase occurred within the last three years, on disease severity as assessed by the machine learning model. This multivariable linear regression was adjusted for age, gender, and smoking status. The coefficients associated with medication purchases, along with their *p*-values, are presented in Table 2.

Spearman correlation analyses were conducted as secondary analyses for medications that were previously identified as significant in the linear regression models to evaluate potential dose–response relationships between the number of doses and relative PD severity. The results of these Spearman correlations are presented along with scatter plots in Figure 3.

Graphs of this study were produced in Python with *seaborn* and *matplotlib* libraries.

### 2.7. Machine Learning Model for Disease Severity Tracking

To track disease severity progression in Parkinson’s disease (PD) patients, we developed a regression-based gradient-boosting model using the LightGBM Python library. The model was trained on a dataset of 8966 patient-years of medication consumption data from 1446 PD patients, with a follow-up period of up to 20 years. No data were excluded from this analysis. The input variables consisted of medication consumption records, provided as yearly quantities for each medication identifier, while the target variable was the number of years since the first diagnosis of PD, serving as a proxy for disease severity.

By training the model to predict disease severity based on medication usage, we aimed to estimate PD progression using yearly medication purchases. To ensure robust evaluation and avoid overfitting, the model was trained using 20-fold cross-validation. All data for a given patient were kept within the same fold during cross-validation to prevent data leakage between training and validation sets, ensuring that past or future data from the same patient were not included in the validation set.

The model hyperparameters were optimized using the FLAML/AutoML framework, which automatically explores the parameter space to improve model performance. A list of the input variables and model hyperparameters is provided in Supplementary Tables S10 and S11.

Disease severity predictions were obtained from the folds that were not trained with the patient’s data. During evaluation, the model demonstrated a strong correlation between predicted disease severity and actual years since diagnosis, with a Pearson correlation coefficient of R = 0.437 (*p* < 10^−110^). This suggests that the model effectively captured relevant patterns in the medication data, providing meaningful predictions of disease severity.

**Figure 2:**
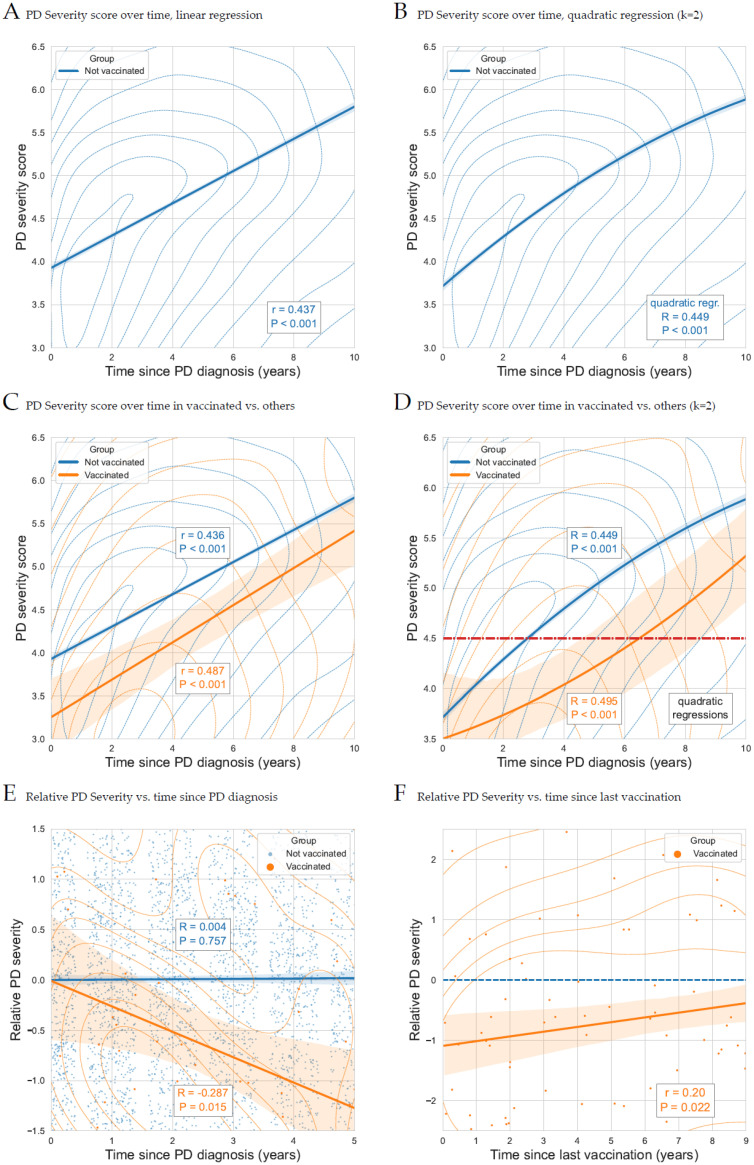
Severity kernel density plots and regression analyses. (**A**) PD severity score over time, kernel density plot and linear regression, (**B**) PD severity score over time, kernel density plot and quadratic regression (k = 2), (**C**) PD severity score over time, kernel density plot and linear regression in vaccinated patients vs. others, (**D**) PD severity score over time, kernel density plot and quadradic regression (k = 2) in vaccinated vs. others, (**E**) relative PD severity over time since PD diagnosis, kernel density plot and linear regression in vaccinated vs. others, and (**F**) relative PD severity vs. time since last vaccination in vaccinated.

**Figure 3:**
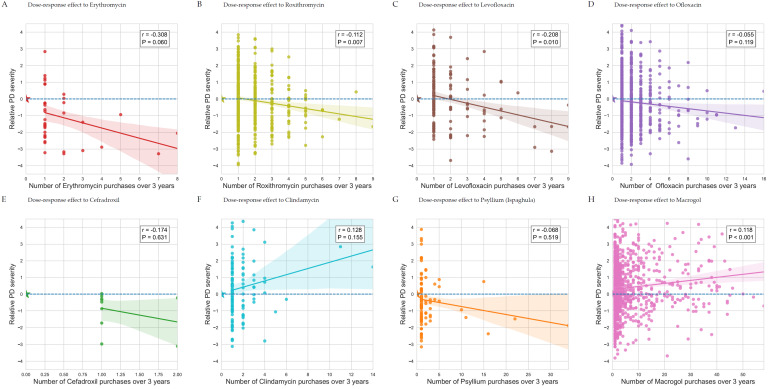
Dose–response effect associated with purchase of selected antimicrobial agents and food supplements. (**A**) Dose–response effect to erythromycin, (**B**) dose–response effect to roxithromycin, (**C**) dose–response effect to levofloxacin, (**D**) dose–response effect to ofloxacin, (**E**) dose–response effect to cefadroxil, (**F**) dose–response effect to clindamycin, (**G**) dose–response effect to psyllium, and (**H**) dose–response effect to macrogol.

Based on these predicted PD severity scores, we define “relative PD severity scores”, which are PD severity scores normalized by the year of disease and which are obtained by subtracting the mean severity score of patients at the same disease year from the severity scores. These relative severity scores are used in the analyses presented in Figure 2E,F and Figure 3.

## 3. Results

### 3.1. The Study Cohort

We performed a large-scale observational study in Leumit Health Services (LHS), a nationwide health organization in Israel with over 22 years of centrally maintained electronic health records (EHR). Using rigorous criteria, we selected 1446 patients who received a PD diagnosis between the ages of 45 and 75, avoiding the rare cases occurring in very young ages (<45), where the disease is heavily influenced by genetic factors, and older patients (>75), where non-specific motor symptoms make the clinical diagnosis of PD less reliable. We used the earliest diagnosis date or antiparkinsonian medication purchase as the index date and selected 7230 control individuals matching in a 5:1 ratio the PD patients. Control individuals were assigned the same index dates as their respective cases so that the same depth of recorded EHR history was available in the two groups.

Table 1 presents the demographic and clinical comparison of the two at the index date. The age, gender, and socioeconomic distribution of the two groups are very similar. In both groups, 54.7% were male, with an average age of 65.7 ± 7.2. Clinical characteristics also appear to be similar, with the notable exception of smoking status, with PD patients being less likely to be smokers: Odds Ratio for current smoking (OR = 0.59; *p* < 0.001), consistent with the literature [16,17].

**Table 1:** Demographic and clinical characteristics of the study cohort at index date.

		PD Patients *N = 1446*	Control *N = 7230*	*p*	Odds Ratio
Gender	Female	655 (45.3%)	3275 (45.3%)	1.000	1
	Male	791 (54.7%)	3955 (54.7%)	1.000	1
Age (years)		65.7 ± 7.2	65.6 ± 7.3	0.537	
Age category	45–49	47 (3.25%)	243 (3.36%)	0.873	0.97
	50–59	247 (17.08%)	1249 (17.28%)	0.879	0.99
	60–69	591 (40.87%)	3081 (42.61%)	0.232	0.93
	70-	561 (38.80%)	2657 (36.74%)	0.136	1.09
Weight (kg)		77.1 ± 15.8	78.1 ± 15.7	0.026	
	Missing	29 (2.01%)	109 (1.51%)		
Height (cm)		165 ± 12	165 ± 11	0.264	
	Missing	53 (3.67%)	182 (2.52%)		
BMI (kg/m^2^)		28.3 ± 5.1	28.6 ± 5.1	0.035	
	Missing	55 (3.80%)	188 (2.60%)		
BMI category	<18.5 Underweight	17 (1.22%)	55 (0.78%)	0.110	1.57
	18.5–24.9 Normal	330 (23.72%)	1618 (22.98%)	0.554	1.04
	25–29.9 Overweight	581 (41.77%)	2951 (41.91%)	0.929	0.99
	≥30 Obese	463 (33.29%)	2418 (34.34%)	0.458	0.95
	Missing	55 (3.80%)	188 (2.60%)	0.014	1.48
BP systolic (mmHg)		134 ± 25	133 ± 21	0.246	
	Missing	4 (0.28%)	41 (0.57%)		
BP diastolic (mmHg)		78.2 ± 9.6	78.0 ± 9.5	0.491	
	Missing	5 (0.35%)	45 (0.62%)		
Smoking status	Non-smoker	1155 (79.88%)	5478 (75.77%)	0.001	1.27
	Past smoker	43 (2.97%)	275 (3.80%)	0.145	0.78
	Smoker	132 (9.13%)	1051 (14.54%)	<0.001	0.59
	Missing	116 (8.02%)	426 (5.89%)	0.003	1.39
Socio-economic status (1–20)		9.72 ± 3.40	9.73 ± 3.40	0.948	
	Missing	74 (5.12%)	370 (5.12%)		
eGFR MDRD (mL/min/1.73 m^2^)		79.4 ± 20.2	80.6 ± 24.5	0.089	
	Missing	132 (9.13%)	844 (11.67%)		
Glucose (mg/dL)		111 ± 32	109 ± 33	0.107	
	Missing	127 (8.78%)	807 (11.16%)		
Hemoglobin A1c (%)		6.22 ± 1.10	6.22 ± 1.16	0.945	
	Missing	418 (28.9%)	2490 (34.4%)		
Record of prior tetanus–diphtheria toxoid vaccination		22 (1.52%)	216 (2.99%)	0.001	0.50

### 3.2. Vaccination Is Associated with Decreased Risk of PD Occurrence

The last line of Table 1 displays a striking difference between the groups for TD vaccination status: only 1.52% of patients with PD had a record of vaccination before the index date, compared to 2.99% in the control group (OR = 0.50, *p* = 0.001).

A characteristic feature of a disease occurring because of waning antibody protection is a progressive increase in the disease rate with time elapsing since vaccination, as our group has shown for SARS-CoV-2 [15,18]. We therefore assessed the relationship between time elapsed since the last vaccination and PD risk.

### 3.3. PD Risk in Vaccinated Patients Is Associated with Time Elapsed Since Vaccination

Figure 1 presents comparisons between the case and control groups based on the timing of vaccination, accompanied by forest plots that graphically display the results. We first compare the proportions of individuals in the case group of Parkinson’s disease (PD) with those in the control group, utilizing the Fisher’s Test (Figure 1A). The odds ratio for developing PD in the absence of recorded vaccination is 1.53 (*p* = 0.018), but this drops to 0.00 within two years after last tetanus–diphtheria (TD) vaccination (*p* = 0.007). These odds increase to 0.16 (*p* = 0.002) within five years and to 0.26 (*p* = 0.004) between five and ten years. Between ten and fifteen years post-vaccination, the reduction in risk loses statistical significance, and after fifteen years, the trend indicates an increased risk, though not statistically significant. Subsequently, we employ logistic regression models (Figure 1B), treating the timing of TD vaccination as a categorical variable with no recorded vaccination as the reference category. This analysis is conducted both with and without adjustments for age, gender, and smoking status as covariates. Both analytical approaches demonstrate a time-dependent protective effect of TD vaccination.

If not receiving a TD vaccination was to be a confounder of some early symptoms of PD (e.g., people with early signs of PD, before diagnosis, may decrease their activity and hence reduce opportunities to become wounded and subsequently receive vaccination), then one would expect that this pattern would continue and even amplify after the index date. However, this does not occur; instead, we observe an opposite trend of increased odds for PD among those who received a vaccine after the index date (OR = 1.33, *p* = 0.39). This reversing time relationship suggests that the lack of TD vaccination is unlikely to be a confounder for early PD.

Having shown that PD occurrence is highly associated with time elapsed since the last TD vaccination, we proceeded to verify, in the few patients who were either diagnosed with PD after vaccination or were vaccinated once PD was diagnosed, whether the disease course was affected by vaccination.

### 3.4. Vaccinated PD Patients Have Slower Rates of Disease Progression

In order to follow the disease course, we developed a method to accurately assess disease severity during the follow-up period. Parkinson’s disease is characterized by a progressive course of motor and functional deterioration, as shown by Hoehn and Yahr’s clinical staging of the laterality and axial symptoms [19] and by the Unified Parkinson’s Disease Rating Scale (UPDRS) [20]. Symptoms are typically treated by antiparkinsonian medications that are increased over time as the disease progresses in dosage and potency.

Having records of medications purchased by each patient, we could use the annual medication consumption to train a machine learning model that would assess disease severity for each patient and year of disease. For this purpose, we trained a gradient boosting model by cross-validation folds, utilizing yearly medication consumption per catalog entry as training variables, together with the gender of the patient (since disease course is affected by gender), to estimate the time elapsed since disease onset, set as the target variable. The output of the model is a PD severity score expressed in a scale analogous to years of disease (e.g., a patient with a severity score of 6 in a given year has purchased medications that are typically used by a PD patient in the 6th year of the disease). Using this model, we computed severity scores for 8793 PD patient-years of disease, of which 201 were from patients with prior vaccination records.

Figure 2A displays a kernel density plot of these scores vs. the actual disease duration, along with a linear regression line. The calculated severity scores correlate nicely with the actual year of the disease. The Pearson correlation coefficient, *r* is 0.437, with a *p*-value under 10^−200^. Of note, a quadratic regression model (a polynomial model of degree 2) was slightly more informative, exhibiting a lower Akaike Information Criterion (AIC) and a higher R^2^ compared to the linear model (see Figure 2B). The associations presented in Figure 2 are also presented as full scatter plots in Figure S1, provided as Supplementary Materials.

Figure 2C displays the severity scores over time, according to vaccination status. Disease severity in vaccinated patients (orange curve) was significantly lower than in non-vaccinated patients. Linear regression models show vaccination status is significantly associated with reduced severity, both in a univariable model (*p* < 0.001, Table S1) and in a multivariable model adjusted for age, gender, and smoking status (*p* < 0.001, Table S2). Here again, the quadratic regression is more informative (Table S3), so we use a quadratic model to plot disease severity according to the time elapsed since PD diagnosis. Figure 2D shows that, even though disease severity was roughly similar at disease onset, disease progression was significantly slower in the vaccinated group. The non-linear disease severity progression suggests two counteracting trends: a natural increase in severity over time and a protective effect of the vaccine, which diminishes with time: the average severity in vaccinated patients at the seventh year of the disease (red line) was similar to the average severity score of unvaccinated patients who were at the third year of the disease.

Figure 2E displays linear regression analysis for the first 5 years of disease data, where the vaccine protection is the strongest. This analysis displays a trend for decreased relative disease severity over time for the vaccinated.

We see here, that even among PD patients, disease course was milder post-vaccination than in PD patients with no prior vaccination record.

### 3.5. Disease Progression Is Associated with Time Elapsed Since Vaccination

We observed that Parkinson’s disease (PD) progresses more slowly in patients who have been vaccinated compared to those who have not. To further investigate whether the TD vaccine affects PD progression, we examined vaccinated patients specifically to determine if the effect is more pronounced closer to the vaccination date, even after adjusting for the time since diagnosis. For this analysis, which requires comparing patients with varying disease durations, we calculated “relative PD severity scores”. These scores were derived by subtracting the mean severity score of patients in the same disease year from individual severity scores, enabling a standardized comparison across different stages of the disease.

Figure 2E displays these relative PD severity scores according to time since diag-nosis, divided by vaccination status in the first five years of this disease. This analysis shows that vaccinated individuals have lower disease severity (*p* < 0.001, Table S4). Figure 2F follows the relative disease severity of vaccinated patients according to the time elapsed since the last vaccination. The relative disease severity decrease is correlated with time elapsed since vaccination (*r* = 0.20, *p* = 0.022, Tables S5 and S6 for crude and adjusted regression analyses).

### 3.6. Disease Severity Is Affected by Antimicrobial Treatments

If *C. tetani* present in the patient’s microbiome plays a role in PD pathology, then PD disease severity should be affected by antimicrobial treatments that affect these bacteria. Having calculated PD disease severity scores, we can use them to assess whether antimicrobial courses are actually associated with changes in disease severity.

For this analysis, we identified forty classes of medications for which we observed varying consumption rates between PD patients and control patients in the years prior to the index date, which is a potential signal for an effect on disease occurrence [21]. For each of these classes, and for each year of disease in a PD patient, we calculated a variable that reflects whether medications of the class were purchased by the patient over the three preceding years. Starting with a multivariable regression model that includes age, gender, and smoking status, we employed a stepwise approach to incrementally select the ten medication classes most strongly associated with the relative PD severity scores. Table 2 presents the results of this regression. Not surprisingly, the tetanus toxoid vaccine had the strongest effect on disease severity, the purchase of the vaccine in the preceding three years significantly reduced the relative disease severity (−1.11, *p* = 0.0003).

**Table 2:** Multivariable linear regression model examining the impact of medication purchased in the three preceding years on relative PD disease severity.

ATC Code		Patient-Years	Coefficient	95% Confidence Interval	*p*-Value
	Age		−0.00	[−0.01 to 0.00]	0.157
	Smoker		0.18	[0.05 to 0.32]	0.006
J01MA01	Ofloxacin	981/8793	−0.45	[−0.55 to −0.34]	<0.0001
A06AD15	Macrogol	999/8793	0.32	[0.22 to 0.43]	<0.0001
J01FA01	Erythromycin	66/8793	−0.73	[−1.11 to −0.35]	0.0002
A06AC01	Ispaghula (psyllium)	184/8793	−0.42	[−0.65 to −0.19]	0.0003
J07AM51	Tetanus toxoid vaccine	26/8793	−1.11	[−1.72 to −0.51]	0.0003
J01CE08	Benzathine benzylpenicillin	46/8793	−0.71	[−1.16 to −0.25]	0.0023
J01DB05	Cefadroxil	21/8793	−1.03	[−1.70 to −0.36]	0.0028
J01FF01	Clindamycin	160/8793	0.39	[0.15 to 0.64]	0.0017
J01FA06	Roxithromycin	1159/8793	−0.14	[−0.24 to −0.04]	0.0055
J01MA12	Levofloxacin	269/8793	−0.23	[−0.42 to −0.04]	0.0193

Several antibiotic agents displayed a substantial and significant effect in reducing disease severity: benzathine penicillin, which is an intramuscular formulation of penicillin, displayed a significant reducing effect on disease severity (−0.71, *p* = 0.002). Benzathine penicillin is the classical treatment for tetanus [22], its sustained-release formulation is likely to deplete the clostridial population producing the toxin, explaining its beneficial effect on disease severity. Likewise, cefadroxil, a first-generation cephalosporine structurally close to penicillin, displayed a significant decreasing effect on disease severity (−1.03, *p* = 0.003). Two macrolide antibiotics also displayed a beneficial effect on disease severity: erythromycin (−0.73, *p* = 0.0002) and roxithromycin (−0.14, *p* = 0.006), as well as two compounds of the fluoroquinolone family: ofloxacin (−0.45, *p* < 0.001), and levofloxacin (−0.23, *p* = 0.019).

On the other hand, clindamycin was associated with a significant and substantial increase in disease severity (+0.39, *p* = 0.002). Clindamycin is primarily used to treat infections caused by susceptible anaerobic bacteria, but it is notorious for causing *Clostridium difficile* colitis, a dangerous condition in which Clostridium bacteria, inherently resistant to clindamycin, colonize the human colon [23]. The observed effect of increasing the severity of PD is consistent with a competitive advantage granted by this antibiotic to clostridia over susceptible anaerobes present in the microbiome, enabling the proliferation of *C. tetani* in a manner similar to *C. difficile* proliferation following clindamycin treatment.

Interestingly, we also observed strong opposing effects for two laxative agents commonly used to treat constipation in PD patients. Macrogol (polyethylene glycol), was significantly associated with increased disease severity (+0.32, *p* < 0.001), while ispaghula (psyllium), a dietary fiber, was associated with decreased disease severity (−0.42, *p* = 0.0003). Interestingly, macrogol is an osmotic laxative that draws water into the colon, increasing stool water content and volume, and creating a more anaerobic environment, which can favor the growth of anaerobic bacteria like Clostridium species. Moreover, experimental studies have shown increased and prolonged clostridium species colonization following polyethylene glycol administration [24]. Conversely, psyllium is a soluble fiber that acts as a probiotic, promoting the growth of beneficial gut bacteria that produce SCFAs like butyrate, which have anti-inflammatory properties and help maintain gut barrier integrity [25]. This environment is less favorable for pathogenic Clostridium proliferation.

We investigated whether a dose–response relationship could be identified by examining the correlation between the number of medication purchases each patient made in the three preceding years and their relative PD severity score. Figure 3 presents scatter plots and correlation analyses for the relevant compounds, acknowledging the limitation that some graphs may exhibit poor fits due to a low number of data points or bins. Nonetheless, for most compounds, we observed a significant dose–response relationship, reinforcing the direction of the association detected by the regression analysis. Since the initial regression only utilized a binary variable to indicate whether each compound had been purchased, without accounting for the number of purchases, identifying a significant dose–response relationship provides additional, independent confirmation of the impact of these medications.

## 4. Discussion

This large-scale, real-life population study shows a negative association between diphtheria-tetanus (DT) toxoid vaccination on both PD risk and its rate of progression. The significant associations observed between time elapsed since vaccination and both PD occurrence and rate of disease progression support the interpretation that the anti-tetanus vaccine provides protection against PD and that this protection wanes over time. In addition, disease progression is significantly associated, in a dose-dependent manner, with antibiotics that affect clostridium growth negatively (beta-lactam, macrolides, fluoroquinolones) or positively (clindamycin), strongly supporting the involvement of actual clostridium bacteria as the causative agent of disease.

Clostridia are known to reside in the microbiome, and several studies have reported differential prevalence of clostridial species in the gut microbiome of PD patients [12,13]. There is indication that *C. tetani* could be commonly present in the human microbiome: a study performed in Vietnam incidentally reported *C. tetani* presence in the feces of 50.0% of 100 patients admitted for tetanus and 42.9% of 28 control subjects [14]. *C. tetani* are frequently present in the saliva, and contamination of a wound with saliva in an unvaccinated individual is an indication for treatment with tetanus immunoglobulin [26]. *C. Tetani* spores are prevalent in the human environment and have extremely resistant properties that should allow this organism to colonize the human flora. *C. tetani* toxin secreted in the gut would primarily affect the mesenteric neurons that regulate peristalsis. Remarkably, impairment of the autonomic nervous system of the gut, manifested as constipation, happens to be a very common prodromal non-motor symptom of PD [27].

After observing Lewy bodies in the mesenteric nervous system and the dorsal nucleus of the vagus nerve, Braak et al. hypothesized that PD pathology begins in the GI tract and further spreads to the CNS [28]. Hawkes and others have proposed a dual-hit mechanism involving two routes: gastrointestinal, through the vagal nerve, and nasal, in which the pathology spreads via anterograde progression into the temporal lobe [29,30].

Remarkably, tetanus neurotoxin injected in the mouse gut has been shown to ascend the vagal nerve [31], and the tetanus toxin is able to reach the basal ganglia via retrograde axonal transport [32]. Colonization of the oropharynx by *C. tetani* could provide a second hit. When present in this niche, *C. tetani* could secrete toxins that would primarily harm the olfactory system, manifesting as smell disturbances [33]. Once significant damage is incurred to dopamine-secreting neurons, toxin-mediated damage may manifest by the characteristic movement disturbances of PD [34], while nonspecific neurodegeneration may present as depression and dementia [35].

Given its prevalence in the environment, exposure to *C. tetani*, even without the neutralizing response provided by vaccination, is certainly not the sole factor of PD occurrence. Genetic and environmental factors, as well as lifestyle and comorbidities, likely influence the ability of *C. tetani* to colonize the microbiome and invade niches from which it could harm adjacent neuronal tissue. Interestingly, cigarette smoking has been shown to inhibit *Clostridium* growth [36]; hence, mere inhibition of *C. tetani* proliferation could explain the decreased risk observed for PD in smokers [16,17]. Being an obligate anaerobe, increased air circulation through the oral cavity would be toxic to *C. tetani* present in the oropharynx. This mechanism could explain some of the protective effects associated with physical activity in PD [37]. Last, it is noteworthy that rural living and farming, which have been associated with increased PD incidence [38], also involve frequent contact with soil, animal feces, and secretions, where *C. tetani* bacilli and spores are widespread. Thus, increased exposure to *C. tetani* may help explain the higher risk of PD observed in this population. 

Our study has some limitations. First, as a database study based on electronic health records documented since 2003, the vaccination history for earlier patients is limited, potentially underreporting true vaccination rates. Second, our study relies on vaccination purchase records from affiliated pharmacies and documented nurse treatment records, potentially missing vaccinations performed outside our health organization, especially in emergency settings. In Israel, tetanus vaccination in adults who had been vaccinated during childhood is primarily administered to individuals with wounds susceptible to tetanus infection rather than as a routine booster. This practice likely contributes to the relatively low vaccination rates observed in our study compared to countries with routine Td vaccination schedules. While individuals employed in specific occupations, such as agricultural or construction work, may be required to undergo vaccination due to their increased exposure, which could act as a confounder related to more strenuous work [39], it is important to note that these occupations represent only a very small portion of the LHS population, which is mostly urban.

Almost all patients in our cohort received a dT formulation combining tetanus toxoid with diphtheria toxoid, an effective adjuvant. This prevents us from ruling out the possibility that the observed effect is associated with diphtheria rather than tetanus vaccination. Our study cohort includes all patients diagnosed with PD between 2003 and 2023 who met the inclusion criteria, totaling 1446 PD patients and 7230 matched control individuals. Future studies with larger, properly powered cohorts are needed to confirm these results and ensure their generalizability.

## 5. Conclusions

This large cohort study demonstrates a significant negative association between combined diphtheria–tetanus (dT) toxoid vaccination and the occurrence of Parkinson’s disease (PD), with an effect that is stronger soon after vaccination. Moreover, among PD patients, using annual antiparkinson medication consumption as a measure of disease severity, we observed slower PD severity progression in patients who had recently received dT vaccination or purchased certain antimicrobial treatments known to target *C. tetani*.

These observations support the hypothesis that *C. tetani* may play a role in the pathological process leading to the progressive neurodegeneration observed in PD. Based on the observed epidemiological associations, we propose that *C. tetani* colonization of the microbiome at sensitive sites may influence PD pathogenesis and progression. Specifically, we suggest that *C. tetani* may contribute to synaptic dysfunction and neurodegeneration through the secretion of its neurotoxin.

These findings suggest that vaccination against tetanus neurotoxin, possibly combined with treatments aimed at eradicating *C. tetani* from bodily reservoirs, may offer promising strategies for preventing PD occurrence and slowing its progression. This hypothesis is testable within the framework of current knowledge: if *C. tetani* is indeed involved in the disease process, vaccination against tetanus neurotoxin, along with antimicrobial treatments that kill *C. tetani* or impair its proliferation, would be expected to slow PD progression and reduce disease occurrence in subjects at risk.

The findings of this study support the need for prospective controlled trials to investigate and confirm the protective effects that anti-tetanus vaccination and antimicrobial treatments may have on PD occurrence and progression. Further research should also explore factors that may influence *C. tetani* proliferation, colonization potential, and cytokine or chemokine responses, particularly in relation to medication, diet, food supplements, hygiene products, and lifestyle habits.

A.I. and E.Me had access to all the original data and served as guarantors of the study’s integrity.

## Data Availability

This study is based on real-world patient data, which are not readily available to external researchers due to LHS IRB restrictions based on patient privacy concerns. Correspondence and requests for materials should be addressed to A.I.

## Supplementary Materials

The following supporting information can be downloaded at: https://www.mdpi.com/article/10.3390/biomedicines12122687/s1, Table S1. Linear regression of severity score vs. time since PD diagnosis; Table S2. Linear regression of severity score vs. time since PD diagnosis adjusted for age, gender, and smoking status; Table S3. Linear regression of severity score vs. time since PD diagnosis (quadratic) adjusted for age, gender, and smoking status; Table S4. Linear regression of relative PD severity vs. vaccination status and time elapsed since PD diagnosis; Table S5. Linear regression of relative PD severity in vaccinated according to time elapsed since vaccination; Table S6. Linear regression of relative PD severity in vaccinated according to time elapsed since vaccination adjusted for covariates; Table S7. List of medications used to determine Parkinson’s Disease; Table S8. List of diagnoses excluding eligibility in the cohort; Table S9. List of antipsychotic medications purchased prior to the index date prevents inclusion in the cohort; Table S10. List of variables included in the disease severity model. For each medication, we compute the number of units purchased each year and the annual score; Table S11. LightGBM model hyperparameters; Figure S1. Scatter plots for kernel density plots displayed in Figure 2.
