# Supplementary material for: Anti-Tetanus Vaccination Is Associated with Reduced Occurrence and Slower Progression of Parkinson’s Disease—A Retrospective Study
Source: Biomedicines. 2024 Nov 25;12(12):2687. doi: 10.3390/biomedicines12122687 (PMC11726988; doi:10.3390/biomedicines12122687)
Supplement: Supplementary file 1 [file biomedicines-12-02687-s001.zip › biomedicines-3248431-supplementary.pdf]

Supplementary Material

Anti-Tetanus Vaccination Is Associated with Reduced Occurrence and Slower Progression of Parkinson's Disease—A Retrospective Study

Ariel Israel [1,2,*], Eli Magen [3], Eytan Ruppin [4], Eugene Merzon [1,5], Shlomo Vinker [1,6] and Nir Giladi [7]

[1] Leumit Research Institute, Leumit Health Services, Tel-Aviv 6473817, Israel
[2] Department of Epidemiology and Preventive Medicine, School of Public Health, Faculty of Medical & Health Sciences, Tel Aviv University, Tel-Aviv 6997801, Israel
[3] Medicine A Department, Assuta Ashdod University Medical Center, Ben Gurion University of the Negev, Beer Sheva 8410501, Israel
[4] Cancer Data Science Laboratory, National Cancer Institute, National Institutes of Health, Bethesda, MD 20814, USA
[5] Adelson School of Medicine, Ariel University, Ariel 4070000, Israel
[6] Department of Family Medicine, Faculty of Medical & Health Sciences, Tel-Aviv 6997801, Israel
[7] Brain Institute, Tel-Aviv Sourasky Medical Center, Faculty of Medical & Health Sciences, Sagol School of Neurosciences, Tel-Aviv University, Tel-Aviv 6997801, Israel
* Correspondence: aisrael@leumit.co.il

The PDF file includes:

Tables S1 to S11

Table S1. Linear regression of severity score vs. time since PD diagnosis.

	Coef	95% Confidence Interval	P. Value
(Intercept)	3.87	[3.82 to 3.92]	0.00000
vaccinated	-0.59	[-0.87 to -0.31]	0.00005
Year since diagnosis	0.19	[0.18 to 0.20]	0.00000

Table S2. Linear regression of severity score vs. time since PD diagnosis adjusted for age, gender, and smoking status.

	Coef	95% Confidence Interval	P. Value
(Intercept)	3.940	[3.63 to 4.25]	0.00000
vaccinated	-0.530	[-0.81 to -0.24]	0.00029
Year since diagnosis	0.190	[0.18 to 0.20]	0.00000
age	0.000	[-0.01 to 0.00]	0.19372
Gender Female	0.270	[0.20 to 0.34]	0.00000
smoker	0.180	[0.05 to 0.32]	0.00878

AIC 8249.830

Table S3. Linear regression of severity score vs. time since PD diagnosis (quadratic) adjusted for age, gender, and smoking status.

	Coef.	[CI]	Pr(>|t|)
(Intercept)	3.760	[3.46 to 4.07]	0.00000
vaccinated	-0.540	[-0.82 to -0.26]	0.00016
Year since diagnosis	0.310	[0.29 to 0.33]	0.00000
I(Year since diagnosis^2)	-0.010	[-0.01 to -0.01]	0.00000
age	0.000	[-0.01 to 0.00]	0.12073
Gender Female	0.280	[0.21 to 0.34]	0.00000
smoker	0.200	[0.07 to 0.34]	0.00290

AIC 8132.973

Table S4. Linear regression of relative PD severity vs. vaccination status and time elapsed since PD diagnosis.

	Coef	[CI]	Pr(>|t|)
(Intercept)	0.010	[-0.04 to 0.06]	0.80900
vaccinated	-0.560	[-0.84 to -0.28]	0.00009
Year since vaccination	0.000	[-0.01 to 0.01]	0.93520

AIC 8057.771

Table S5. Linear regression of relative PD severity in vaccinated according to time elapsed since vaccination.

	Coef	[CI]	Pr(>|t|)
(Intercept)	-1.060	[-1.61 to -0.51]	0.00024
Year since vaccination	0.070	[0.01 to 0.14]	0.03447

AIC 129.9

Table S6. Linear regression of relative PD severity in vaccinated according to time elapsed since vaccination adjusted for covariates.

	Coef	[CI]	Pr(>|t|)
(Intercept)	9.060	[5.33 to 12.80]	0.00001
Year since vaccination	0.070	[0.00 to 0.13]	0.05090
Year since diagnosis	0.130	[0.05 to 0.21]	0.00147
age	-0.150	[-0.20 to -0.09]	0.00000
Gender Female	-0.850	[-1.60 to -0.10]	0.02805
smoker	-2.650	[-4.17 to -1.12]	0.00091

AIC 110.15

Table S7. List of medications used to determine Parkinson's Disease.

N04AA04	Procyclidine
N04BA02	Levodopa and Decarbo
N04BA03	Levodopa, Decarboxyl
N04BA07	foslevodopa and deca
N04BB01	Amantadine
N04BC02	Pergolide
N04BC04	Ropinirole
N04BC05	Pramipexole
N04BC07	Apomorphine
N04BD01	Selegiline
N04BD02	Rasagiline
N04BD03	Safinamide
N04BX02	Entacapone

Table S8. List of diagnoses excluding eligibility in the cohort.

ICD9 code	Diagnosis description
225.11	ADENOMA OF HYPOPHYSE
227.3	ADENOMA OF HYPOPHYSE-BENIGN NEOPLASM OF PITUITARY GLAND AND CRANIOPHARYNGEAL DUCT
253.12	PROLACTINOMA
253.120	MICROADENOMA OF HYPOPHYSE
253.121	MACROADENOMA OF HYPOPHYSE
295	SCHIZOPHRENIC DISORDERS
295.0	SIMPLE TYPE SCHIZOPHRENIA
295.00	SIMPLE TYPE SCHIZOPHRENIA, UNSPECIFIED STATE
295.01	SIMPLE TYPE SCHIZOPHRENIA, SUBCHRONIC STATE
295.02	SIMPLE TYPE SCHIZOPHRENIA, CHRONIC STATE
295.03	SIMPLE TYPE SCHIZOPHRENIA, SUBCHRONIC STATE WITH ACUTE EXACERBATION
295.04	SIMPLE TYPE SCHIZOPHRENIA, CHRONIC STATE WITH ACUTE EXACERBATION
295.05	SIMPLE TYPE SCHIZOPHRENIA, IN REMISSION
295.1	DISORGANIZED TYPE SCHIZOPHRENIA
295.10	DISORGANIZED TYPE SCHIZOPHRENIA, UNSPECIFIED STATE
295.11	DISORGANIZED TYPE SCHIZOPHRENIA, SUBCHRONIC STATE
295.12	DISORGANIZED TYPE SCHIZOPHRENIA, CHRONIC STATE
295.13	DISORGANIZED TYPE SCHIZOPHRENIA, SUBCHRONIC STATE WITH ACUTE EXACERBATION
295.14	DISORGANIZED TYPE SCHIZOPHRENIA, CHRONIC STATE WITH ACUTE EXACERBATION
295.15	DISORGANIZED TYPE SCHIZOPHRENIA, IN REMISSION
295.2	CATATONIC TYPE SCHIZOPHRENIA
295.20	CATATONIC TYPE SCHIZOPHRENIA, UNSPECIFIED STATE
295.21	CATATONIC TYPE SCHIZOPHRENIA, SUBCHRONIC STATE
295.22	CATATONIC TYPE SCHIZOPHRENIA, CHRONIC STATE
295.24	CATATONIC TYPE SCHIZOPHRENIA, CHRONIC STATE WITH ACUTE EXACERBATION
295.25	CATATONIC TYPE SCHIZOPHRENIA, IN REMISSION
295.3	PARANOID TYPE SCHIZOPHRENIA
295.30	PARANOID TYPE SCHIZOPHRENIA, UNSPECIFIED STATE
295.31	PARANOID TYPE SCHIZOPHRENIA, SUBCHRONIC STATE
295.32	PARANOID TYPE SCHIZOPHRENIA, CHRONIC STATE
295.33	PARANOID TYPE SCHIZOPHRENIA, SUBCHRONIC STATE WITH ACUTE EXACERBATION
295.34	PARANOID TYPE SCHIZOPHRENIA, CHRONIC STATE WITH ACUTE EXACERBATION
295.35	PARANOID TYPE SCHIZOPHRENIA, IN REMISSION
295.4	ACUTE SCHIZOPHRENIC EPISODE
295.40	ACUTE SCHIZOPHRENIC EPISODE, UNSPECIFIED STATE
295.41	ACUTE SCHIZOPHRENIC EPISODE, SUBCHRONIC STATE

295.42	ACUTE SCHIZOPHRENIC EPISODE, CHRONIC STATE
295.43	ACUTE SCHIZOPHRENIC EPISODE, SUBCHRONIC STATE WITH ACUTE EXACERBATION
295.44	ACUTE SCHIZOPHRENIC EPISODE, CHRONIC STATE WITH ACUTE EXACERBATION
295.45	ACUTE SCHIZOPHRENIC EPISODE, IN REMISSION
295.5	SCHIZOTYPAL DISORDER-LATENT SCHIZOPHRENIA
295.50	SCHIZOTYPAL DISORDER-LATENT SCHIZOPHRENIA, UNSPECIFIED STATE
295.52	SCHIZOTYPAL DISORDER-LATENT SCHIZOPHRENIA, CHRONIC STATE
295.53	SCHIZOTYPAL DISORDER-LATENT SCHIZOPHRENIA, SUBCHRONIC STATE WITH ACUTE EXACERBATION
295.54	SCHIZOTYPAL DISODER-LATENT SCHIZOPHRENIA, CHRONIC STATE WITH ACUTE EXACERBATION
295.55	SCHIZOTYPAL DISORDER-LATENT SCHIZOPHRENIA, IN REMISSION
295.6	RESIDUAL SCHIZOPHRENIA
295.60	RESIDUAL SCHIZOPHRENIA, UNSPECIFIED STATE
295.62	RESIDUAL SCHIZOPHRENIA, CHRONIC STATE
295.64	RESIDUAL SCHIZOPHRENIA, CHRONIC STATE WITH ACUTE EXACERBATION
295.65	RESIDUAL SCHIZOPHRENIA, IN REMISSION
295.7	SCHIZO-AFFECTIVE TYPE SCHIZOPHRENIA
295.70	SCHIZO-AFFECTIVE TYPE SCHIZOPHRENIA, UNSPECIFIED STATE
295.71	SCHIZO-AFFECTIVE TYPE SCHIZOPHRENIA, SUBCHRONIC STATE
295.72	SCHIZO-AFFECTIVE TYPE SCHIZOPHRENIA, CHRONIC STATE
295.73	SCHIZO-AFFECTIVE TYPE SCHIZOPHRENIA, SUBCHRONIC STATE WITH ACUTE EXACERBATION
295.74	SCHIZO-AFFECTIVE TYPE SCHIZOPHRENIA, CHRONIC STATE WITH ACUTE EXACERBATION
295.75	SCHIZO-AFFECTIVE TYPE SCHIZOPHRENIA, IN REMISSION
295.8	OTHER SPECIFIED TYPES OF SCHIZOPHRENIA
295.80	OTHER SPECIFIED TYPES OF SCHIZOPHRENIA, UNSPECIFIED STATE
295.82	OTHER SPECIFIED TYPES OF SCHIZOPHRENIA, CHRONIC STATE
295.83	OTHER SPECIFIED TYPES OF SCHIZOPHRENIA, SUBCHRONIC STATE WITH ACUTE EXACERBATION
295.84	OTHER SPECIFIED TYPES OF SCHIZOPHRENIA, CHRONIC STATE WITH ACUTE EXACERBATION
295.85	OTHER SPECIFIED TYPES OF SCHIZOPHRENIA, IN REMISSION
295.9	UNSPECIFIED SCHIZOPHRENIA
295.90	UNSPECIFIED TYPE SCHIZOPHRENIA, UNSPECIFIED STATE
295.91	UNSPECIFIED TYPE SCHIZOPHRENIA, SUBCHRONIC STATE
295.92	UNSPECIFIED TYPE SCHIZOPHRENIA, CHRONIC STATE
295.920	OBSESSIVE-COMPULSIVE DISORDER, UNSPECIFIED
295.93	UNSPECIFIED TYPE SCHIZOPHRENIA, SUBCHRONIC STATE WITH ACUTE EXACERBATION
295.94	UNSPECIFIED TYPE SCHIZOPHRENIA, CHRONIC STATE WITH ACUTE EXACERBATION

332.1	PARKINSONISM, SECONDARY
332.10	PARKINSONISM DUE TO DRUGS
332.11	TARDIVE DYSKINESIA
333.99	RESTLESS LEGS
333.990	PERIODIC LEGS MOVEMENTS
431	INTRACEREBRAL HEMORRHAGE
434.01	CEREBRAL THROMBOSIS WITH CEREBRAL INFARCTION
434.11	CEREBRAL EMBOLISM WITH CEREBRAL INFARCTION
434.9	CEREBRAL ARTERY OCCLUSION, UNSPECIFIED
434.90	ISCHEMIC CEREBROVASCULAR DISEASE (CVA) WITHOUT MENTION OF INFARCTION
434.91	ISCHEMIC CEREBROVASCULAR DISEASE (CVA) WITH INFARCTION
436	ACUTE, BUT ILL-DEFINED, CEREBROVASCULAR DISEASE-CVA
436.3	STROKE
431	INTRACEREBRAL HEMORRHAGE
434.01	CEREBRAL THROMBOSIS WITH CEREBRAL INFARCTION
434.11	CEREBRAL EMBOLISM WITH CEREBRAL INFARCTION
434.9	CEREBRAL ARTERY OCCLUSION, UNSPECIFIED
434.90	ISCHEMIC CEREBROVASCULAR DISEASE (CVA) WITHOUT MENTION OF INFARCTION
434.91	ISCHEMIC CEREBROVASCULAR DISEASE (CVA) WITH INFARCTION
436	ACUTE, BUT ILL-DEFINED, CEREBROVASCULAR DISEASE-CVA
436.3	STROKE

Table S9. List of antipsychotic medications which purchase prior to the index date prevents inclusion in the cohort.

N05AA01	Chlorpromazine
N05AA02	Levomepromazine
N05AB02	Fluphenazine
N05AB03	Perphenazine
N05AB06	Trifluoperazine
N05AB08	Thioproperazine
N05AC01	Periciazine
N05AC02	Thioridazine
N05AD01	Haloperidol
N05AE03	Sertindole
N05AE04	Ziprasidone
N05AF01	Flupentixol
N05AF03	Chlorprothixene
N05AF05	Zuclopenthixol
N05AG02	Pimozide
N05AG03	Penfluridol
N05AH02	Clozapine
N05AH03	Olanzapine
N05AH04	Quetiapine
N05AH05	Asenapine
N05AH06	Clotiapine
N05AL01	Sulpiride
N05AL03	Tiapride
N05AL05	Amisulpride
N05AN01	Lithium
N05AX08	Risperidone
N05AX12	Aripiprazole
N05AX13	Paliperidone
N05AX14	Iloperidone
N05AX15	Cariprazine
N05AX16	Brexpiprazole

Table S10. List of variables included in the disease severity model. For each medication we compute the number of units purchased each year the annual score.

**Gender of the patient (1 for female)**				
Medication (yearly quantity of medication unit for each of the following catalog entries)	ATC code	ATC description	Serving Form	Giving Form
AMANDIN 100 MG 100 TAB	N04BB01	Amantadine	TB	PO
AMANTADIN HX 100MG 30 29G	N04BB01	Amantadine	TB	PO
AMANTADINE 100MG 100T 29G	N04BB01	Amantadine	TB	PO
AMANTADINSULFAT 200MG_500	N04BB01	Amantadine	IJ	IV
APO-GO 10 MG/ML 5ML 5 AMP	N04BC07	Apomorphine	IJ	SC
APO-GO 20 MG/2ML 5 AMP	N04BC07	Apomorphine	IJ	SC
APO-GO PEN 10 MG_ML 3ML*5	N04BC07	Apomorphine	SR	SC
AZILECT 1 MG 30 TAB	N04BD02	Rasagiline	TB	PO
COMTAN 200 MG 30 TAB	N04BX02	Entacapone	TB	PO
DEKINET 2 MG 30 TAB	N04AA02	Biperiden	TB	PO
DOPICAR 30 TAB	N04BA02	Levodopa and Decarbo	TB	PO
DUODOPA GEL 100 ML 7 CASS	N04BA02	Levodopa and Decarbo	GE	IS
ENTACAPONE 200 MG 30 TAB	N04BX02	Entacapone	TB	PO
JUMEX 5 50 TAB	N04BD01	Selegiline	TB	PO
KEMADRIN 5 MG 100 TAB	N04AA04	Procyclidine	TB	PO
LEVOPAR PLUS 125 MG 30 C	N04BA02	Levodopa and Decarbo	TB	PO
LEVOPAR PLUS 250 MG 30 C	N04BA02	Levodopa and Decarbo	TB	PO
MADOPAR 100/25MG CAP 29-G	N04BA02	Levodopa and Decarbo	CP	PO
MADOPAR 200/50MG C MBI 29	N04BA02	Levodopa and Decarbo	CP	PO
MADOPAR 200/50MG CAP 29-G	N04BA02	Levodopa and Decarbo	CP	PO
PARILAC 10 MG 20 CAP	N04BC01	Bromocriptine	CP	PO
PARITREL 20 TAB	N04BB01	Amantadine	TB	PO
PARTANE 2 MG 50 TAB	N04AA01	Trihexyphenidyl	TB	PO
PARTANE 5 MG 50 TAB	N04AA01	Trihexyphenidyl	TB	PO

PERGOLIDE 0.05 MG 100 TA	N04BC02	Pergolide	TB	PO
PERGOLIDE 0.25 MG 100 T	N04BC02	Pergolide	TB	PO
PERGOLIDE TEVA 0.25MG 100	N04BC02	Pergolide	TB	PO
PK MERZ 100 MG TAB 29-G	N04BB01	Amantadine	TB	PO
PK MERZ 100 TAB 29-G	N04BB01	Amantadine	TB	PO
PK MERZ 100MG TAB RAZ 29G	N04BB01	Amantadine	TB	PO
PK MERZ INF 200MG/500ML*2	N04BB01	Amantadine	IJ	IV
PRAMIPEXOLE TEVA 0.25 MG	N04BC05	Pramipexole	TB	PO
PRAMIPEXOLE TEVA 1MG 100T	N04BC05	Pramipexole	TB	PO
RASAGILINE-TRIMA 1 MG 30	N04BD02	Rasagiline	TB	PO
REQUIP 0.25 MG 21 TAB	N04BC04	Ropinirole	TB	PO
REQUIP 0.25 MG 210 TAB	N04BC04	Ropinirole	TB	PO
REQUIP 0.5 MG 21 TAB	N04BC04	Ropinirole	TB	PO
REQUIP 1 MG 21 TAB	N04BC04	Ropinirole	TB	PO
REQUIP 1 MG 84 TAB	N04BC04	Ropinirole	TB	PO
REQUIP 2 MG 21 TAB	N04BC04	Ropinirole	TB	PO
REQUIP 2 MG 84 TAB	N04BC04	Ropinirole	TB	PO
REQUIP 5 MG 21 TAB	N04BC04	Ropinirole	TB	PO
REQUIP 5 MG 84 TAB	N04BC04	Ropinirole	TB	PO
REQUIP MODUTAB 2 MG 28TAB	N04BC04	Ropinirole	TB	PO
REQUIP MODUTAB 4 MG 28TAB	N04BC04	Ropinirole	TB	PO
REQUIP MODUTAB 8 MG 28TAB	N04BC04	Ropinirole	TB	PO
RODENAL 2 MG 30 TAB	N04AA01	Trihexyphenidyl	TB	PO
RODENAL 5 MG 30 TAB	N04AA01	Trihexyphenidyl	TB	PO
ROPINIROLE 0.25 MG 210 T	N04BC04	Ropinirole	TB	PO
ROPINIROLE 0.5 MG 21 TAB	N04BC04	Ropinirole	TB	PO
ROPINIROLE 1 MG 84 TAB	N04BC04	Ropinirole	TB	PO
ROPINIROLE 2 MG 84 TAB	N04BC04	Ropinirole	TB	PO

ROPINIROLE 5 MG 84 TAB	N04BC04	Ropinirole	TB	PO
SELEGILINA GENER 5MG 29-G	N04BD01	Selegiline	TB	PO
SELEGILINE 5 MG 50 T CTS	N04BD01	Selegiline	TB	PO
SIFROL 0.25 MG 100 TAB	N04BC05	Pramipexole	TB	PO
SIFROL 1.0 MG 100 TAB	N04BC05	Pramipexole	TB	PO
SIFROL ER 0.375 MG 30 TAB	N04BC05	Pramipexole	TB	PO
SIFROL ER 0.75 MG 30 TAB	N04BC05	Pramipexole	TB	PO
SIFROL ER 1.5 MG 30 TAB	N04BC05	Pramipexole	TB	PO
SINEMET CR 30 TAB	N04BA02	Levodopa and Decarbo	TB	PO
SINEMET CR 50_200 100 TAB	N04BA02	Levodopa and Decarbo	TB	PO
SINEMET CR 50_200 60T 29G	N04BA02	Levodopa and Decarbo	TB	PO
SINEMET RETARD 50/200 100	N04BA02	Levodopa and Decarbo	TB	PO
STALEVO 100/25/200 MG 30T	N04BA03	Levodopa, Decarboxyl	TB	PO
STALEVO 125/31.25/200 30T	N04BA03	Levodopa, Decarboxyl	TB	PO
STALEVO 150/37.5/200MG 30	N04BA03	Levodopa, Decarboxyl	TB	PO
STALEVO 200/50/200 MG 30T	N04BA03	Levodopa, Decarboxyl	TB	PO
STALEVO 50/12.5/200 MG 30	N04BA03	Levodopa, Decarboxyl	TB	PO
STALEVO 75/18.75/200MG 30	N04BA03	Levodopa, Decarboxyl	TB	PO
TRIMEXOL 0.25 MG 100 TAB	N04BC05	Pramipexole	TB	PO
TRIMEXOL 1 MG 100 TAB	N04BC05	Pramipexole	TB	PO
XADAGO 100 MG 30 TAB	N04BD03	Safinamide	TB	PO
XADAGO 50 MG 30 TAB	N04BD03	Safinamide	TB	PO

Table S11. LightGBM model hyperparameters.

Model hyperparameters
Objective: Regression
Metric: Root Mean Squared Error (RMSE)
Number of estimators: 3,108
Number of leaves: 45
Minimum child samples: 2
Learning rate: 0.00196
Max bin: 127
Colsample by tree: 0.424
Regularization (Alpha): 0.0020
Regularization (Lambda): 14.98

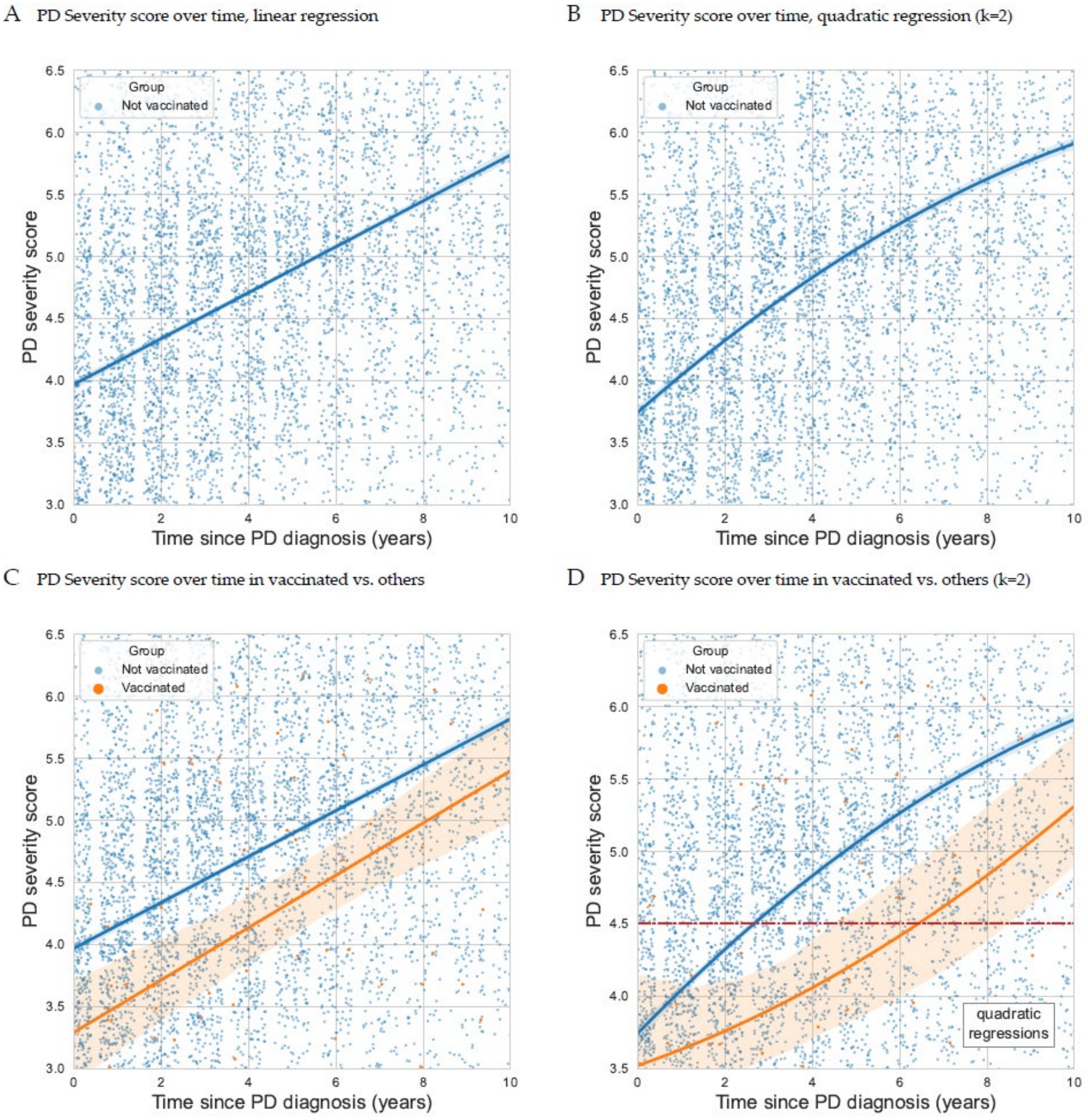

Figure S1. Scatter plots for kernel density plots displayed in Figure 2.
